# Is foraging innovation lost following colonization of a less variable environment? A case study in surface‐ vs. cave‐dwelling *Asellus aquaticus*

**DOI:** 10.1002/ece3.6276

**Published:** 2020-05-05

**Authors:** Gábor Herczeg, Viktória P. Hafenscher, Gergely Balázs, Žiga Fišer, Simona Kralj‐Fišer, Gergely Horváth

**Affiliations:** ^1^ Behavioural Ecology Group Department of Systematic Zoology and Ecology Biological Institute Eötvös Loránd University Budapest Hungary; ^2^ Department of Biology Biotechnical Faculty University of Ljubljana Ljubljana Slovenia; ^3^ Institute of Biology Research Centre of the Slovenian Academy of Sciences and Arts Ljubljana Slovenia

**Keywords:** adaptation, behavioral flexibility, behavioral innovation, colonization, plasticity

## Abstract

Behavioral innovation is a key process for successful colonization of new habitat types. However, it is costly due to the necessary cognitive and neural demands and typically connected to ecological generalism. Therefore, loss of behavioral innovativeness is predicted following colonization of new, simple, and invariable environments. We tested this prediction by studying foraging innovativeness in the freshwater isopod *Asellus aquaticus*. We sampled its populations along the route of colonizing a thermokarstic water‐filled cave (simple, stable habitat with only bacterial mats as food) from surface habitats (variable environment, wide variety of food). The studied cave population separated from the surface populations at least 60,000 years ago. Animals were tested both with familiar and novel food types (cave food: bacterial mats; surface food: decaying leaves). Irrespective of food type, cave individuals were more likely to feed than surface individuals. Further, animals from all populations fed longer on leaves than on bacteria, even though leaves were novel for the cave animals. Our results support that cave *A. aquaticus* did not lose the ability to use the ancestral (surface) food type after adapting to a simple, stable, and highly specialized habitat.

## Graphical abstract

We showed that cave‐adapted freshwater isopod *Asellus aquaticus* did not lose its ability to recognize and even prefer surface food after at least 60,000 years of isolation  in the cave . This result suggest that adaptation to a highly specialized and stable habitat does not necessary include a decrease in behavioral innovativeness.

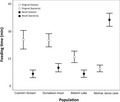

## 1 INTRODUCTION

Behavioral innovation is an individual's invented new behavior or modified old behavior not present previously in the population (Reader, 2003; Reader & Laland, 2003), which seems to be a key asset for success in novel situations and thus a prerequisite of successful colonization of new habitat types (Mayr, 1965; Morse, 1980). It is a process that results in novel behaviors either in the sense of technical innovations (novel mechanism) or the use of novel resources (these two can work independently; Overington, Morand‐Ferron, Boogert, & Lefebvre, 2009). The typical approaches in understanding variation in behavioral innovativeness include comparative evolutionary analyses based on phylogenetically corrected interspecific comparisons (e.g., Ducatez, Clavel, & Lefebvre, 2015; Garamszegi, Erritzoe, & Møller, 2007; Overington, Griffin, Sol, & Lefebvre, 2011; Overington et al., 2009; Reader & Laland, 2002) or within‐population experimental studies (e.g., Laland & Reader, 1999; Taylor, Elliffe, Hunt, & Gray, 2010).

Comparative studies revealed a number of correlations with behavioral innovativeness, for instance, showing its positive link to brain size and learning capacity (e.g., Lefebvre, Reader, & Sol, 2004; Overington et al., 2009; Reader & Laland, 2002). These are interesting results supporting the idea that cognition and innovativeness require relatively large brains (Buechel, Boussard, Kotrschal, Bijl, & Kolm, 2018; Collado, Montaner, Molina, & Sol, 2020; Sol, Lefebvre, & Rodríguez‐Teijeiro, 2005). But what sort of environments select for increased innovativeness? One potential benefit of behavioral innovation is the ability to exploit a wider range of resources (Reader & MacDonald, 2003), which suggests that higher innovativeness is expected in ecological generalists. Overington et al. (2011) tested this idea on 193 North American bird species and found a positive correlation between habitat generalism and innovation rate. The correlations listed above can explain why innovative, large‐brained ecological generalists are more likely to successfully colonize new habitats (Sol, Duncan, Blackburn, Cassey, & Lefebvre, 2005; Sol & Lefebvre, 2000; Sol, Timmermans, & Lefebvre, 2002). It has also been repeatedly shown that generalists are likely to have larger brains and better learning abilities than specialists, because generalists need to process more environmental information (Daly, Rauschenberger, & Behrends, 1982; Ratcliffe, Fenton, & Shettleworth, 2006). However, all studies mentioned in this paragraph are based on phylogenetically corrected interspecific comparisons. Such studies can be seen as the cornerstones of understanding evolutionary adaptation, but they suffer from the inherent difficulty in separating causation from correlation (Gonda, Herczeg, & Merilӓ, 2013). On the other hand, intraspecific studies, particularly between‐population comparisons, are scarce at best (Reader, 2003). This is unfortunate, because understanding adaptive evolution of quantitative traits relies on estimating (a) phenotypic variation, (b) selection acting on the variation, (c) the heritable component of the variation, and ultimately, (d) the genetic underpinnings of the variation. These goals can be achieved in intraspecific studies. The logical first step in this process is to compare phenotypes between populations adapted to their different selective environments. Therefore, comparing behavioral innovativeness between locally adapted populations of the same species would be an important addition to understanding the evolution of behavioral innovation.

A comparative study on birds suggested a positive link between behavioral innovativeness and habitat generalism (Overington et al., 2011). Testing this hypothesis in an intraspecific context would require a species where populations inhabit environments with different levels of isolation and spatial or temporal variation. Behavioral innovation can be seen as a special form of (behavioral) phenotypic plasticity, which is a genotype's ability to express/develop alternative phenotypes (e.g., West‐Eberhard, 2003). Similarly to various forms of phenotypic plasticity, behavioral innovation is not only beneficial, but also involves various costs (DeWitt, Sih, & Wilson, 1998). These costs can be broadly divided into costs of (a) expression and (b) developing and maintaining the machinery (brain, sensory organs) needed to gather and process environmental information. For behavioral plasticity, the latter costs are dominant (Snell‐Rood, 2013), especially in our case (see below). Therefore, populations inhabiting isolated, simple, and stable habitats where the benefit of behavioral innovation is minimal are expected to lose their costly innovativeness during the course of local adaptation. It is noteworthy that most studies on behavioral innovation are done on mammals and birds because these taxa usually exhibit higher cognitive abilities. In most invertebrates, however, simpler forms of behavioral innovation are expected. Overington et al. (2009) distinguished two foraging innovation types, (i.e.) technical and food type (resource) innovation, and the latter is definitely expected to be important across all animal taxa. We note that food type innovation in invertebrates might not require high cognitive performance, but it is still expected to be linked to energetically costly sensory and neural systems needed for perception as well as decision and choice.

In the present paper, we aimed to test whether a habitat and food generalist species would decrease food type innovation after adapting to a new environment with reduced habitat complexity and negligible food type diversity. Our model, the freshwater isopod *Asellus aquaticus*, is widespread in a wide variety of surface freshwater habitats across the Western Palearctic (Verovnik, Sket, & Trontelj, 2004, 2005) and has successfully colonized caves in Europe on several independent occasions (Verovnik, Prevorčnik, & Jugovic, 2009; Verovnik & Konec, 2019). These cave‐adapted populations are typically sustained by organic matter coming from the surface. Here, we focused on an *A. aquaticus* population from a particular thermokarstic water‐filled cave (Molnár János Cave, Hungary), which is unique in the sense that organic material from the surface (e.g., logs, leaves, algae, soil) does not enter the cave and the only apparent food resource for the resident isopods is cave‐growing bacteria forming mats. This is in stark contrast with the typical surface populations of *A. aquaticus*, which consume various forms of living and dead plant material (e.g., Bloor, 2011; Graça, Maltby, & Calow, 1993; Moore, 1975). The Molnár János Cave population is not physically separated from the neighboring surface populations due to the constant water outflow into a pond that is connected to a river. Nevertheless, the cave population shows adaptations typical for cave life (eye degeneration and body depigmentation). Further, based on mitochondrial and nuclear markers, this cave population diverged from the surface populations at least 60,000 years ago (Pérez‐Moreno, Balázs, Wilkins, Herczeg, & Bracken‐Grissom, 2017).

We compared the feeding behavior of *A. aquaticus* from one cave and three surface populations: (a) the Molnár János Cave, (b) a thermal pond formed by the water outflow at the entrance of the cave, (c) a nearby stream, and (d) a nearby moor. All individuals were tested against food sources that were either native or unknown in their original habitat (native in cave: bacterial mats; native in surface: decaying leaves). We predicted that the cave population adapted to consume the bacterial mats will not be able to switch to decaying leaves, while the generalist surface populations will readily consume the bacterial mats. In other words, we predicted that adaptation to the cave environment involves a shift from feeding generalism toward specialization including the loss of food type innovativeness. We note that the environment of the three surface populations was quite different, but they were similar in terms of high variability of potential food sources and in the lack of the cave food type. Therefore, we predicted no difference among the surface populations in food type innovativeness. In addition, we also tested the general hypothesis that populations adapted to negligible predation will show elevated behavioral activity (e.g., Bell, 2005; Herczeg, Gonda, & Merilӓ, 2009; Magurran & Seghers, 1991). Here, we predicted that cave *A. aquaticus* living in a habitat without predators will display higher feeding activity.

## 2 MATERIAL AND METHODS

### 2.1 Molnár János Cave

To be confident about interpreting the outcome of our study, we need to be sure that *A. aquaticus* in the Molnár János Cave are not meeting with decaying leaves (or any exogenous food source) at all. The occurrence of particular organic matter of external origin in the cave is practically impossible for the following reasons. It has been shown that cave genesis in the study area is driven by the mixing corrosion effect of ascending thermal and lukewarm waters (Kovács & Müller, 1980; Mádl‐Szőnyi, Erőss, & Tóth, 2017); therefore, the Molnár János Cave is a hypogene cave (i.e., water forming the cave did not come from the surface; Erőss, 2010; Klimchouk, 2007). Further, the effects of infiltrating descending waters (through porous limestone bedrock and perhaps tiny fissures in the bedrock) are negligible (Leél‐Össy et al. 2011). In accordance with this, Total Organic Carbon could only be detected in extremely low amounts in the waters of Molnár János Cave (Dobosy, Sávoly, Óvári, Mádl‐Szonyi, & Záray, 2016). Finally, during hundreds of dives over a decade in the cave, a single visible piece of organic material of surface origin was never observed, but cave *A. aquaticus* were routinely seen feeding on the bacterial mats (G. Balázs, personal observation). Therefore, we think that this cave's community is entirely based on organic material produced by chemoautotrophic bacteria living in darkness, similar to the Movile Cave in Romania (Sarbu, Kane, & Kinkle, 1996). We are aware that this claim is yet unproven, but we are confident that food common to surface *A. aquaticus* populations is entirely absent from the cave population's diet (apart from potentially consuming cave animals’ carcasses). We note that the cave community is extremely simple and predators of *A. aquaticus* are completely absent in Molnár János Cave.

### 2.2 Sampled populations

We used one cave and three surface populations of the freshwater isopod *A. aquaticus*. All populations live within or in close surroundings of Budapest, Hungary. The cave population dwells in Molnár János Cave and shows typical troglomorph adaptations, such as eye degeneration and depigmentation. The cave population has been genetically isolated from surface populations (including the Malom Lake population, see below) for at least 60,000 years, despite the absence of physical barriers and occasional surface individuals entering the cave (Pérez‐Moreno et al., 2017). Molnár János Cave is a thermokarstic water‐filled cave and *A. aquaticus* can be found in areas where water temperature is between 23 and 24°C all year round. The only apparent food source for this cave population of *A. aquaticus* is bacterial mats (see above). The cave's outflow to the surface forms a small lake (Malom Lake) right at the cave entrance (47.518277° N, 19.035999° E), harboring the first sampled surface population. Malom Lake is subjected to the natural surface light regime of the region, but the water temperature is similar to the one in the cave all year round. Apart from the constant water temperature, this habitat can be seen as a typical surface habitat. It is connected to the river Danube. The remaining two surface populations, i.e. the Csömöri Stream (47.593393° N, 19.121970° E) and the Dunakeszi Peat‐moor (47.615613° N, 19.126392° E), experience natural surface light regime and temperature fluctuations typical to the region. These populations were chosen randomly, representing typical surface habitats. All three surface populations live in habitats with diverse communities and various food sources, such as algae, living and dead plant material together with fungal and bacterial overgrow.

### 2.3 Collecting and housing the experimental animals

Adult animals (*N* = 200; 25 males and 25 females per population) were collected between 16 and 17 August 2018. Cave diving was necessary to collect cave individuals. During this time of the year, water temperature at the surface localities was similar to the temperature of thermal water at Malom Lake and Molnár János Cave, that is, 23–24°C. After collection, animals were immediately transported to the facilities of the Biological Institute of Eötvös Loránd University (Budapest, Hungary). All animals were housed individually in 90 × 25 mm plastic Petri dishes. The bottom of all Petri dishes was coarsened with emery paper to enable animals’ normal movement (Fišer, Prevorčnik, Lozej, & Trontelj, 2019). Water collected at the source habitats was used to fill the Petri dishes to the half of their height and was regularly refilled as the water level dropped. Petri dishes with animals were then placed in light‐controlled “recording chambers” (see below) and kept there during the whole experiment (i.e., acclimation and video recording). Surface populations were acclimated in a daily light cycle (16h light : 8h dark), while cave animals were acclimated in complete darkness. All manipulations of cave animals were done under red light. The temperature in the laboratory was 23–24°C. Animals did not receive any food apart from the food provided in feeding tests (see below). As some animals died during the first few days in the laboratory, we eventually tested 163 individuals (Molnár János Cave: 14 males (M) / 16 females (F), Malom Lake: 25 M / 24 F, Csömöri Stream: 20 M / 20 F; Dunakeszi Peat‐moor: 20 M / 24 F).

### 2.4 Experimental setup

To video‐record animal behavior in feeding tests in different light conditions, we used four similar custom made recording chambers (length: 100 cm, width: 55 cm, height: 105 cm). All recording chambers were equipped with two light sources: LEDs imitating daylight (4,500 K, CRI > 90) at the top and infrared LEDs (920 nm) at the bottom. Daylight was switched on only for assaying the surface populations but not for the cave population. Infrared light was switched on during all tests, since it was needed for video recording. Opal plexiglass was placed over the infrared LEDs to evenly diffuse the emitted light and at the same time to serve as a surface on which Petri dishes with animals were put. Each recording chamber could house a maximum of 50 animals. The chambers were closed from sides with nontransparent black plastic boards so that light did not scatter outside of the chamber. Inside each chamber, we mounted a webcam (Logitech C920 FullHD) that was technically modified to improve the quality of videos recorded in infrared light. OBS Studio software (OBS Studio Contributors) was used to capture videos at 5 frames per second at HD resolution (1,280 × 720).

### 2.5 Experimental design and protocol

After collection, all experimental animals were acclimated under their natural light regime without any disturbance and food. On 24 August, we ran the first round of feeding tests followed by two days of rest and the second round of feeding tests on 27 August. The acclimation period before the first test ensured that animals got familiar with their artificial environment and they were eager to feed. The first feeding test likely did not satiate the test animals because they were more likely to feed in the second then in the first test (see Results). This way, we saw a feeding attempt in ca. 70% of the individuals. Feeding tests were done in the given population's natural light regime. All individuals within population were randomly divided into two groups (sexes represented equally). The first group was offered their familiar food source in the first feeding test and the novel food source in the second feeding test, while the second group was first offered the novel and then the familiar food source. We used two food sources: decaying poplar (*Populus* sp.) leaves (familiar to the surface populations) and bacterial mats from the cave (familiar to the cave population). We used rubber rings (diameter: 5 mm, height: 1 mm) in the Petri dishes holding the experimental animals to standardize the position of the provided food and to prevent its dislocation during the recordings. Immediately prior recordings, we filled the food source into the rubber rings. Then, we video‐recorded the animals’ behavior for 60 min. All recordings started at approximately 11 a.m. After the recordings, we removed the remaining food from the Petri dishes.

We used three behavioral variables to describe feeding behavior: (a) “drive to feed” (whether an individual fed in the given test or not), (b) “feeding duration” (total time spent with the food, including both food handling and eating), and (c) “feeding bouts” (number of times an individual approached the food item). A feeding event began when the animal started to process the provided food (judged by body orientation and touching the food source) and ended when the animal moved away a distance equal to its body length. Only individuals that fed were included in the analysis of feeding duration and feeding bouts.

### 2.6 Statistical analyses

Testing our main question about food type innovation was straightforward: we could simply observe whether a test animal consumed the unfamiliar food item or not. However, based on population and treatment effects, we could also test for additional patterns in the above three variables. To analyze drive to feed, we used a generalized linear mixed model (GLMM) with a binomial distribution and a logit link function. In this model, drive to feed was included as a binary response variable (presence vs. absence of feeding), while food type (familiar, novel), population (Molnár János Cave, Malom Lake, Csömöri Stream, Dunakeszi Peat‐moor), sex (male, female), and all their two‐ and three‐way interactions were added as categorical fixed effects. We controlled for habituation to the laboratory setting by adding the centered order of trials as a fixed effect. Individual identity was added to the model as a random effect. We also considered random slopes (i.e., individual × habituation interaction), but we left the random slopes term in the final model only if it improved the model fit. Feeding duration and feeding bouts were analyzed using GLMMs with negative binomial distribution and log link function; fixed and random effects were the same as in the previous model. Error distribution and link function applied in the GLMMs were chosen after inspection of Q‐Q plots of the model residuals. Fixed effects were tested by Wald's chi‐squared tests and random effects by likelihood ratio tests. *P* values for the likelihood ratio tests were calculated following Zuur, Ieno, Walker, Saveliev, and Smith (2009). We note that neither sex, nor its interactions were significant in any of the models. Leaving these predictors out of the models did not change the results qualitatively; hence, we report the full models (Forstmeier & Schielzeth, 2011). All GLMMs were built using the R packages lme4 and lmerTest (Bates, Maechler, Bolker, & Walker, 2015; Kuznetsova, Brockhoff, & Christensen, 2016). All analyses were run in R 3.6.1 (R Developmental Core Team, 2019).

## 3 RESULTS

**FIGURE 1:**
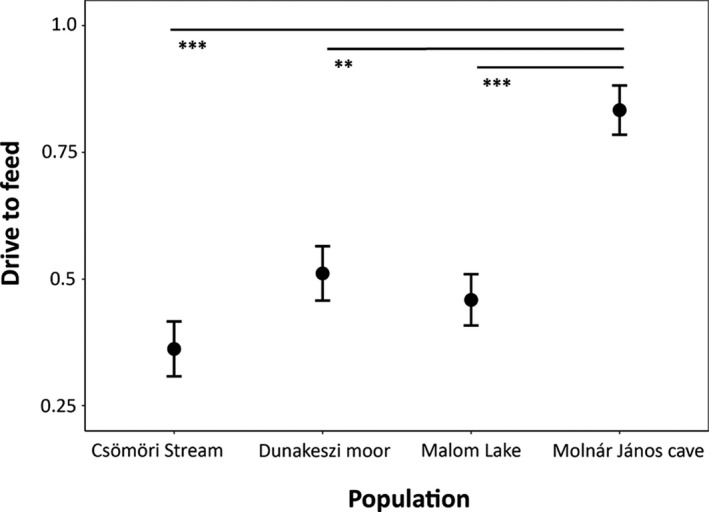
Drive to feed in the four tested populations of *Asellus aquaticus* (significant population effect). Means ± standard errors are shown. Significant post hoc pairwise differences are also shown (Tukey test; ** denotes *p* < .01, while *** *p* < .001).

Out of 163 individuals, 48 did not feed at all, irrespective of food type. Out of the 326 feeding tests, there were 157 cases without feeding. The results of binomial GLMM implied that drive to feed significantly varied between the four populations (Table 1; Figure 1). Specifically, the cave‐adapted *A. aquaticus* were more likely to feed, irrespective of food source, than their surface‐adapted conspecifics. We also found a marginally significant effect of habituation (Table 1). Namely, animals were more likely to feed in the second than in the first trial (data not shown). Individual differences in drive to feed were marginally nonsignificant, but cannot be ruled out (Table 1).

**TABLE 1:** Results of models for drive to feed, feeding duration and feeding bouts of *Asellus aquaticus*. Significant effects are in bold font. Nonsignificant individual × habituation interactions are shown here, but were removed from the final models.

Model term	Drive to feed		Feeding duration		Feeding bouts	
	*χ* ^2^ (*df*)	*p*‐value	*χ* ^2^ (*df*)	*p*‐value	*χ* ^2^ (*df*)	*p*‐value
Fixed effects						
Population	21.82 (3)	**<.001**	15.15 (3)	**.002**	6.37 (3)	.09
Sex	0.46 (1)	.49	0.07 (1)	.79	0.03 (1)	.86
Food type	<0.001 (1)	.99	5.05 (1)	**.03**	2.19 (1)	.14
Population × sex	4.19 (3)	.24	3.55 (3)	.32	1.73 (3)	.63
Population × food type	2.33 (3)	.51	9.76 (3)	**.02**	2.37 (3)	.50
Sex × food type	1.14 (1)	.29	0.002 (1)	.97	0.36 (1)	.55
Population × sex ×food type	2.29 (3)	.51	4.16 (3)	.25	5.74 (3)	.12
Habituation	3.89 (1)	.05	5.16 (1)	**.02**	2.55 (1)	.11
Random effects						
Individual	2.62 (1)	.052	109,918.3 (1)	**<.001**	<0.001 (1)	.5
Individual × habituation	<0.001 (1)	.99	91,229 (1)	**<.001**	3.74 (1)	.10

**FIGURE 2:**
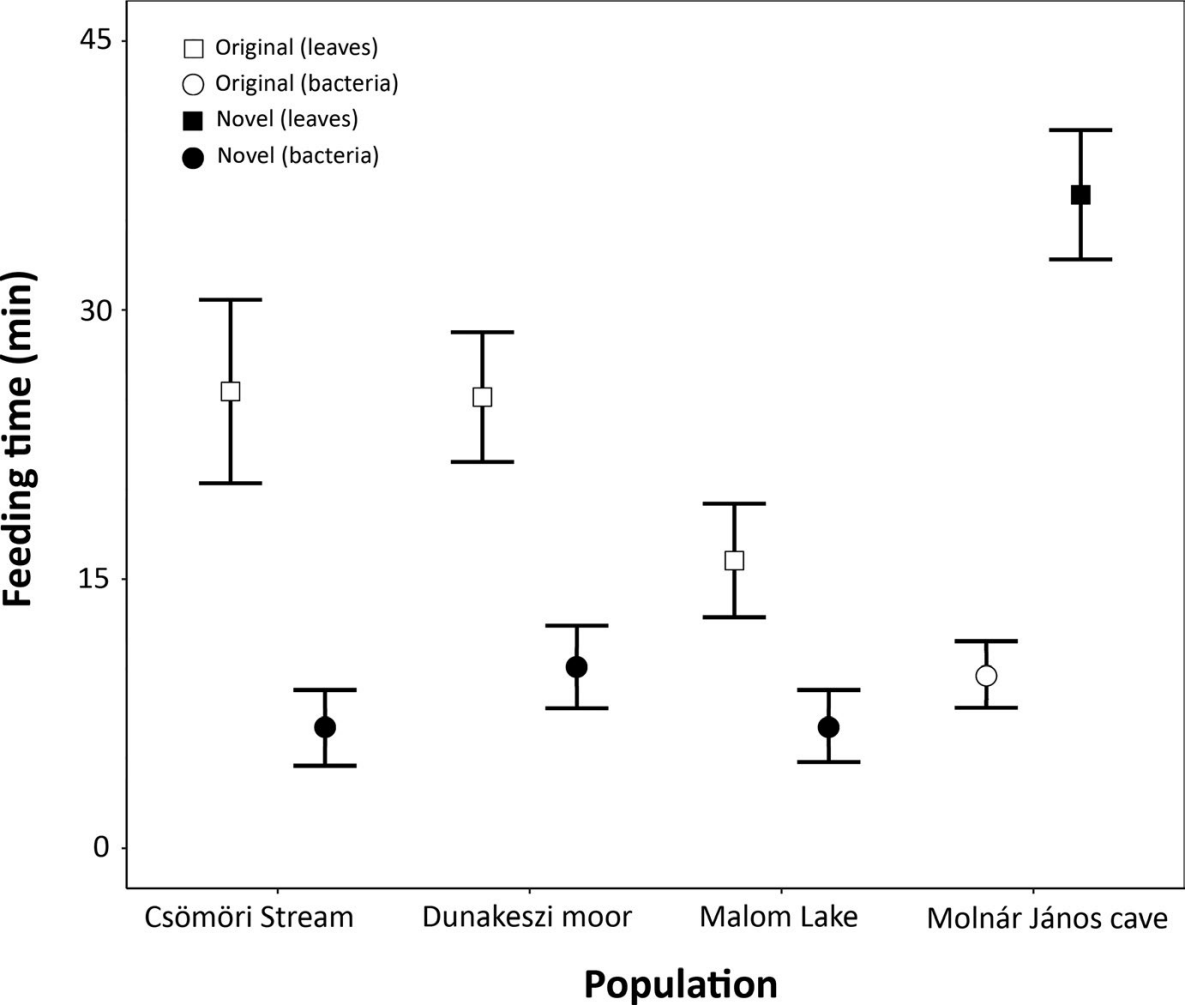
Feeding duration on familiar and novel food types in the four tested populations of *Asellus aquaticus* (significant population × food type interaction). Means ± standard errors are shown.

The first negative binomial GLMM revealed a significant population × food type interaction effect on feeding duration (Table 1; Figure 2): cave‐adapted *A. aquaticus* fed longer on the novel, while surface‐adapted populations fed longer on the familiar food. In other words, animals of all populations spent more time feeding on decaying poplar leaves than on bacterial mats, irrespective of whether it was included in their natural diet or it was novel for them. We also found a significant effect of habituation (Table 1): animals spent more time feeding in the second than in the first trial (data not shown). Further, individual behavioral trends differed significantly (Table 1). We found individuals differing significantly in the duration of feeding (Table 1). The second negative binomial GLMM on feeding bouts revealed no significant effect (Table 1).

## 4 DISCUSSION

Behavioral innovation is regarded as a key process for successful colonization of novel habitat types (e.g., Mayr, 1965; Morse, 1980). However, evolutionary analyses of behavioral innovation are almost exclusively based on interspecific comparisons, where separating causation from correlation is notoriously hard. Therefore between‐population comparisons within the same species are warranted. Further, while behavioral innovation is beneficial for allowing the exploitation of a wider array of resources, it is also costly due to the demand for the development of the energetically costly sensory and nervous system (e.g., Aiello & Wheeler, 1995; Kotrschal et al., 2013). Therefore, an evolutionary loss of innovativeness is expected upon colonizing a new habitat where the benefits of innovativeness are diminished or lost. We tested this hypothesis in a system where generalist surface individuals of *A. aquaticus* successfully colonized a thermokarstic water‐filled cave offering a simple and stable, but highly specialized environment with only one food source that is unknown to surface individuals. Surface *A. aquaticus* is known to consume a variety of food sources, from algae to fungi growing on decaying leaves (e.g., Bloor, 2011; Graça et al., 1993; Moore, 1975), while the only apparent food source in the cave is bacterial mats. We predicted that surface *A. aquaticus* will be able to switch to the cave food type (albeit preferring the surface food type), while cave *A. aquaticus* will not be able to use the surface food type. Contrary to our prediction, all populations fed on both food types and animals of all populations preferred the surface food type. This implies that cave *A. aquaticus* has maintained the ability to recognize and consume surface food, and in fact shows preference for surface food. We note that we interpreted feeding duration as a measure of preference, but alternative explanations are also possible. For instance, it sounds intuitively realistic that the herein studied cave *A. aquaticus* evolved a feeding apparatus that is specialized to consuming bacteria, and thus, it took more time for them to process decaying leaves. However, in a preliminary morphological analysis, we did not find any apparent differences between the mouthparts of the different populations (unpublished data).

We did not detect the predicted evolutionary change in food type innovation (sensu Overington et al., 2009). In other words, we found no support for our prediction that food generalism in surface populations has eventually changed toward food specialization in the cave population, but our data suggest an evolutionary rigid generalist feeding strategy. It is noteworthy that cave *versus* surface populations differed in their feeding time and not in the number of feeding bouts, suggesting that the observed difference is not confounded by general activity. There are at least two potential explanations for these results. First, it is possible that the costs of food type innovation are negligible. For instance, *A. aquaticus* individuals are constantly sampling the environment and simply consume anything edible. However, this strategy still relies on the machinery needed to separate edible from nonedible matter (sensory apparatus and brain), so it does not convincingly explain the reported pattern. Alternatively, it is possible that food type innovation is not lost during the course of evolution due to some proximate constraints. For instance, the genetic and physiological background of the development of the costly sensory and neural machinery might be involved in other vital processes. However, adaptation to the cave environment can include the loss of entire sensory systems like the eye or complex physiological functions like the circadian rhythm (McGaugh et al., 2014; Protas, Trontelj, & Patel, 2011; Yoshizawa et al., 2015), so this argument is not too convincing either. Perhaps the machinery has other important functions than food choice, for instance, in social communication or in orientation. Further experiments are needed to test these alternative explanations.

Besides testing for food type innovation, we also analyzed drive to feed. Here, we found that cave *A. aquaticus* are more likely to initiate feeding irrespective of food type than their surface‐dwelling conspecifics. Higher feeding activity has its obvious benefits (increased energy uptake) and costs (increased exposure to predation). Since the cave community in focus is extremely simple and lacks predators, we predicted increased feeding activity for cave *A. aquaticus*, and our data fully support this prediction. Similar population patterns of increased behavioral activity under negligible predatory risk were found in several studies (e.g., Magurran & Seghers, 1991; Bell, 2005; Herczeg et al., 2009; but see Brown, Jones, & Braithwaite, 2005; Brown, Burgess, & Braithwaite, 2007). However, there is an alternative, but nonexclusive explanation too: in caves, food availability is typically low compared to surface environments (Culver & Pipan, 2009). Therefore, unit energy uptake needs higher behavioral activity directed towards finding food in caves. In support of this explanation, there are several cave species that display increased food‐searching activity (Hüppop, 2000). Further, the well‐studied evolutionary model *Astyanax mexicanus* cavefish exhibits increased appetite and increased locomotor activity coupled with “sleep loss” compared to their close surface relatives (e.g., Aspiras, Rohner, Martineau, Borowsky, & Tab in, 2015; Jeffery, 2001; Yoshizawa et al., 2015). However, food scarcity as a general environmental stressor in caves is true for caves relying on exogenous food sources. In our case, the food source is the endogenous bacterial mats, which can be found in large quantities and might have higher nitrogen and calorific content than plant material (e.g., Prochazka, Payne, & Mayberry, 1970; Smith & Palmer, 1976; Vanbeveren, Gebauer, Plichta, Volařík, & Ceulemans, 2016).

*A. aquaticus* is known for its ability to colonize cave habitats and such colonizations occurred on several independent occasions (Verovnik & Konec, 2019). However, most of the colonized cave systems’ communities are sustained by organic matter coming from the surface. There are only two caves inhabited by *A. aquaticus* known so far where organic matter is not of surface origin: the Molnár János Cave (present study) and the Movile Cave in Romania (Sarbu et al., 1996). We tried to repeat the study presented here in the Movile Cave, but due to certain logistic problems, data gathered during that attempt could not be tested with scientific rigor; therefore, we decided not to present it. However, we note that patterns observed in Movile Cave were different to what we report here. Therein, cave‐dwelling *A. aquaticus* preferred the bacterial mats collected in the cave over surface food. Further, Mösslacher and Creuzé des Châtelliers (1996) showed that surface *A. aquaticus* collected in Austria spent more time feeding than cave *A. aquaticus* from the Movile Cave system when provided with surface food type. However, in both our and Mösslacher and Creuté des Châtelliers’ (1996) studies, *A. aquaticus* from Movile Cave did feed on surface food. Therefore, our findings about preference toward surface food based on the cave population from Molnár János Cave cannot be generalized. However, *A. aquaticus* from both Molnár János Cave and Movile Cave could identify surface food as food, providing examples where the ability to switch food types is maintained even after long isolation with only one main food source.

Taken together, we found that the generalist‐opportunistic feeding strategy of *Asellus aquaticus* did not change after being isolated for at least 60,000 years in a highly specialized and stable habitat with only one food type (absent in other habitats) being available. This finding suggests that (a) food type innovation has only negligible costs in this species or (b) there are developmental, genetic, or physiological constraints acting against the evolutionary change of this trait. To further test our hypothesis, future efforts should aim to target food preference and the reaction to food that is novel to all populations, to quantify behavioral plasticity and behavioral innovation on the individual level to assess its evolvability, and to investigate feeding behavior in the Movile Cave in more detail.

## AUTHOR CONTRIBUTIONS

**Gábor Herczeg:** Conceptualization (equal); Formal analysis (supporting); Funding acquisition (lead); Methodology (equal); Resources (lead); Supervision (lead); Writing‐original draft (lead); Writing‐review & editing (lead). **Viktória P. Hafenscher:** Conceptualization (equal); Formal analysis (supporting); Investigation (equal); Methodology (equal); Writing‐original draft (supporting); Writing‐review & editing (supporting). **Gergely Balázs:** Conceptualization (equal); Investigation (equal); Methodology (equal); Writing‐original draft (supporting); Writing‐review & editing (supporting). **Žiga Fišer:** Conceptualization (equal); Investigation (equal); Methodology (equal); Writing‐original draft (supporting); Writing‐review & editing (supporting). **Simona Kralj‐Fišer:** Conceptualization (equal); Investigation (equal); Methodology (equal); Writing‐original draft (supporting); Writing‐review & editing (supporting). **Gergely Horváth:** Conceptualization (equal); Data curation (lead); Formal analysis (lead); Investigation (equal); Methodology (equal); Supervision (supporting); Writing‐original draft (supporting); Writing‐review & editing (supporting).

## COMPETING INTERESTS

The authors have no competing interests to declare.

## Data Availability

The data are available on DRYAD (https://doi.org/10.5061/dryad.1g1jwstrz).

## References

[ece36276-bib-0001] Aiello, L. C. , & Wheeler, P. (1995). The expensive tissue hypothesis – The brain and digestive system in human and primate evolution. Current Anthropology, 36, 199–221.

[ece36276-bib-0002] Aspiras, A. C. , Rohner, N. , Martineau, B. , Borowsky, R. L. , & Tabin, C. J. (2015). Melanocortin 4 receptor mutations contribute to the adaptation of cavefish to nutrient‐poor conditions. Proceedings of the National Academy of Sciences, 112, 9668–9673. 10.1073/pnas.1510802112 PMC4534248 26170297

[ece36276-bib-0003] Bates, D. , Maechler, M. , Bolker, B. , & Walker, S. (2015). Fitting linear mixed‐ effects models using lme4. Journal of Statistical Software, 67, 1–48.

[ece36276-bib-0004] Bell, A. M. (2005). Behavioural differences between individuals and two populations of stickleback (*Gasterosteus aculeatus*). Journal of Evolutionary Biology, 18, 464–473. 10.1111/j.1420-9101.2004.00817.x 15715852

[ece36276-bib-0005] Bloor, M. C. (2011). Dietary preference of *Gammarus pulex* and *Asellus aquaticus* during a laboratory breeding programme for ecotoxicological studies. International Journal of Zoology, 2011, 1–5.

[ece36276-bib-0006] Brown, C. , Burgess, F. , & Braithwaite, V. A. (2007). Heritable and experiential effects on boldness in a tropical poeciliid. Behavioural Ecology Sociobiology, 62, 237–243. 10.1007/s00265-007-0458-3

[ece36276-bib-0007] Brown, C. , Jones, F. , & Braithwaite, V. A. (2005). In situ examination of boldness‐shyness traits in the tropical poeciliid, *Brachyraphis episcope* . Animal Behaviour, 70, 1003–1009.

[ece36276-bib-0008] Buechel, S. D. , Boussard, A. , Kotrschal, A. , van der Bijl, W. , & Kolm, N. (2018). Brain size affects performance in a reversal‐learning test. Proceedings of the Royal Society B, 285, 20172031 10.1098/rspb.2017.2031 29367391 PMC5805926

[ece36276-bib-0009] Collado, M. Á. , Montaner, C. M. , Molina, F. P. , Sol, D. , & Bartomeus, I. (2020). Brain size predicts learning abilities in bees. bioRxiv. 10.1101/2020.01.27.921114 PMC8131939 34017597

[ece36276-bib-0010] Culver, D. , & Pippan, T. (2009). The biology of caves and other subterranean habitats. Oxford: Oxford University Press.

[ece36276-bib-0011] Daly, M. , Rauschenberger, J. , & Behrends, P. (1982). Food aversion learning in kangaroo rats – a specialist‐generalist comparison. Animal Learning and Behavior, 10, 314–320. 10.3758/BF03213716

[ece36276-bib-0012] DeWitt, T. J. , Sih, A. , & Wilson, D. S. (1998). Costs and limits of phenotypic plasticity. Trends in Ecology and Evolution, 13, 77–81. 10.1016/S0169-5347(97)01274-3 21238209

[ece36276-bib-0013] Dobosy, P. , Sávoly, Z. , Óvári, M. , Mádl‐Szonyi, J. , & Záray, Gy. (2016). Microchemical characterization of biogeochemical samples collected from the Buda Thermal Karst System, Hungary. Microchemical Journal, 124, 116–120. 10.1016/j.microc.2015.08.004

[ece36276-bib-0014] Ducatez, S. , Clavel, J. , & Lefebvre, L. (2015). Ecological generalism and behavioural innovation in birds: Technical innovation or the simple incorporation of new foods? Journal of Animal Ecology, 84, 79–89. 24910268 10.1111/1365-2656.12255

[ece36276-bib-0015] Erőss, A. (2010). Characterization of fluids and evaluation of their effects on karst development at the Rózsadomb and Gellért Hill, Buda Thermal Karst, Hungary.PhD Dissertation, Eötvös Lóránd University, Budapest, 171 pp.

[ece36276-bib-0016] Fišer, Ž. , Prevorčnik, S. , Lozej, N. , & Trontelj, P. (2019). No need to hide in caves: Seeking behavior of surface and cave ecomorphs of *Asellus aquaticus* (Isopoda: Crustacea). Zoology, 134, 58–65. 31146907 10.1016/j.zool.2019.03.001

[ece36276-bib-0017] Forstmeier, W. , & Schielzeth, H. (2011). Cryptic multiple hypothesis testing in linear models: Everestimated effect sizes and the winner’s curse. Behavioural Ecology and Sociobiology, 65, 47–55. 10.1007/s00265-010-1038-5 PMC3015194 21297852

[ece36276-bib-0018] Garamszegi, L. Z. , Erritzoe, J. , & Møller, A. P. (2007). Feeding innovations and parasitism in birds. Biological Journal of the Linnean Society, 90, 441–455. 10.1111/j.1095-8312.2007.00733.x

[ece36276-bib-0019] Gonda, A. , Herczeg, G. , & Merilӓ, J. (2013). Evolutionary ecology of intraspecific brain size variation: A review. Ecology and Evolution, 3, 2751–2764. 10.1002/ece3.627 24567837 PMC3930043

[ece36276-bib-0020] Graça, M. A. S. , Maltby, L. , & Calow, P. (1993). Importance of fungi in the diet of *Gammarus pulex* and *Asellus aquaticus* I. feeding strategies. Oecologia, 93, 139–144. 10.1007/BF00321203 28313786

[ece36276-bib-0021] Herczeg, G. , Gonda, A. , & Merilӓ, J. (2009). Predation mediated population divergence in complex behaviour of nine‐spined stickleback (*Pungitius pungitius*). Journal of Evolutionary Biology, 22, 544–552. 19210595 10.1111/j.1420-9101.2008.01674.x

[ece36276-bib-0022] Hüppop, K. (2000). How do cave animals cope with the food scarcity in caves? In Wilkens H., Culver D. C., & Humphreys F. W. (Eds.), Ecosystems of the world: Subterranean ecosystems (pp. 159–188). Amsterdam: Elsevier.

[ece36276-bib-0023] Jeffery, W. R. (2001). Cavefish as a model system in evolutionary developmental biology. Developmental Biology, 231, 1–12. 10.1006/dbio.2000.0121 11180948

[ece36276-bib-0024] Klimchouk, A. (2007). Hypogene speleogenesis: Hydrogeological and morphogenetic perspective. Special Paper No.1, National Cave and Karst Research Institute, Carlsbad, NM, 106 pp.

[ece36276-bib-0025] Kotrschal, A. , Rogell, B. , Bundsen, A. , Svensson, B. , Zajitschek, S. , Brännström, I. , … Kolm, N. (2013). Artificial selection on relative brain size in the guppy reveals costs and benefits of evolving larger brains. Current Biology, 23, 168–171. 23290552 10.1016/j.cub.2012.11.058 PMC3566478

[ece36276-bib-0026] Kovács, J. , & Müller, P. (1980). A Budai‐hegyek hévizes tevékenységének kialakulása és nyomai. Karszt És Barlang, 2, 93–98 (in Hungarian).

[ece36276-bib-0027] Kuznetsova, A. , Brockhoff, P. B. , & Christensen, R. H. B. (2016).lmerTest: tests in linear mixed effcts models, R package version 2.0–33. Retrieved from https://CRAN.R‐project.org/package=lmerTest

[ece36276-bib-0028] Laland, K. L. , & Reader, S. M. (1999). Foraging innovation in the guppy. Animal Behaviour, 57, 331–340. 10.1006/anbe.1998.0967 10049472

[ece36276-bib-0055] Leél‐Össy, S. Z. , Bergmann, C. S. , & Bognár, C. S. (2011). A budapesti Molnár János‐barlang termálvizének veszélyeztetettsége. A Miskolci Egyetem Közleménye, A Sorozat, Bányászat, 81, 91–102. (in Hungarian).

[ece36276-bib-0029] Lefebvre, L. , Reader, S. M. , & Sol, D. (2004). Brains, innovations and evolution in birds and primates. Brain, Behaviour and Evolution, 63, 233–246. 10.1159/000076784 15084816

[ece36276-bib-0030] Mádl‐Szőnyi, J. , Erőss, A. , & Tóth, Á. (2017). Fluid flow systems and hypogene karst of the transdanubian range, Hungary – With special emphasis on Buda thermal karst In Klimchouk A., Palmer A. N., Waele J. D., & Audra P. (Eds.), Hypogene karst regions and caves of the world (pp. 267–278). Cham: Springer.

[ece36276-bib-0031] Magurran, A. E. , & Seghers, B. H. (1991). Variation in schooling and aggression amongst guppy (*Poecilia reticulata*) populations in Trinidad. Behaviour, 118, 214–234. 10.1163/156853991X00292

[ece36276-bib-0032] Mayr, E. (1965). The nature of colonising birds In Baker H. G., & Stebbins G. L. (Eds.), The genetics of colonising species (pp. 29–43). New York: Academic Press.

[ece36276-bib-0033] McGaugh, S. E. , Gross, J. B. , Aken, B. , Blin, M. , Borowsky, R. , Chalopin, D. , … Warren, W. C. (2014). The cavefish genome reveals candidate genes for eye loss. Nature Communications, 5, 5307 10.1038/ncomms6307 PMC4218959 25329095

[ece36276-bib-0034] Moore, J. W. (1975). The role of algae in the diet of *Asellus aquaticus* L. and *Gammarus pulex* L. Journal of Animal Ecology, 44, 719–730. 10.2307/3714

[ece36276-bib-0035] Morse, D. H. (1980). Behavioural mechanisms in ecology. Cambridge, MA: Harvard University Press.

[ece36276-bib-0036] Mösslacher, F. , & Creuzé des Châtelliers, M. (1996). Physiological and behavioural adaptations of an epigean and a hypogean dwelling population of *Asellus aquaticus* (L.) (Crustacea, Isopoda). Archiv Für Hydrobiologie, 138, 187–198.

[ece36276-bib-0037] Overington, S. E. , Griffin, A. S. , Sol, D. , & Lefebvre, L. (2011). Are innovative species ecological generalists? A test in North American birds. Behavioral Ecology, 22, 1286–1293. 10.1093/beheco/arr130

[ece36276-bib-0038] Overington, S. E. , Morand‐Ferron, J. , Boogert, N. J. , & Lefebvre, L. (2009). Technical innovations drive the relationship between innovativeness and residual brain size in birds. Animal Behaviour, 78, 1001–1010. 10.1016/j.anbehav.2009.06.033

[ece36276-bib-0039] Pérez‐Moreno, J. L. , Balázs, G. , Wilkins, B. , Herczeg, G. , & Bracken‐Grissom, H. D. (2017). The role of isolation on contrasting phylogeographic patterns in two cave crustaceans. BMC Evolutionary Biology, 17, 247 10.1186/s12862-017-1094-9 29216829 PMC5721366

[ece36276-bib-0040] Prochazka, G. J. , Payne, W. J. , & Mayberry, W. R. (1970). Calorific content of certain bacteria and fungi. Journal of Bacteriology, 104, 646–649. 10.1128/JB.104.2.646-649.1970 5489431 PMC285040

[ece36276-bib-0041] Protas, M. E. , Trontelj, P. , & Patel, N. H. (2011). Genetic basis of eye and pigment loss in the cave crustacean, *Asellus aquaticus* . Proceedings of the National Academy of Sciences, 108, 5702–5707. 10.1073/pnas.1013850108 PMC3078413 21422298

[ece36276-bib-0042] R Developmental Core Team . (2019). R: A language and environment for statistical computing. Vienna: R Foundation for Statistical Computing Retrieved from http://www.R‐project.org/

[ece36276-bib-0043] Ratcliffe, J. M. , Fenton, M. B. , & Shettleworth, S. J. (2006). Behavioural flexibility positively correlated with relative brain volume in predatory bats. Brain, Behaviour and Evolution, 67, 165–176. 10.1159/000090980 16415571

[ece36276-bib-0044] Reader, S. M. (2003). Innovation and social learning: Individual variation and brain evolution. Animal Biology, 53, 147–158. 10.1163/157075603769700340

[ece36276-bib-0045] Reader, S. M. , & Laland, K. L. (2002). Social intelligence, innovation and enhanced brain size in primates. PNAS, 99, 4436–4441. 10.1073/pnas.062041299 11891325 PMC123666

[ece36276-bib-0046] Reader, S. M. , & Laland, K. L. (2003). Animal innovation. Oxford, UK: Oxford University Press.

[ece36276-bib-0047] Reader, S. M. , & MacDonald, K. (2003). Environmental variability and primate behavioural flexibility In Reader S. M., & Laland K. L. (Eds.), Animal innovation (pp. 83–116). Oxford, UK: Oxford University Press.

[ece36276-bib-0048] Sarbu, S. M. , Kane, T. C. , & Kinkle, B. K. (1996). A chemoautotrophically based cave ecosystem. Science, 272, 1953–1955. 10.1126/science.272.5270.1953 8662497

[ece36276-bib-0049] Smith, R. H. , & Palmer, R. (1976). A chemical and nutritional evaluation of yeasts and bacteria as dietary protein sources for rats and pigs. Journal of the Science of Food and Agriculture, 27, 763–770. 10.1002/jsfa.2740270810 966729

[ece36276-bib-0050] Snell‐Rood, E. C. (2013). An overview of the evolutionary causes and consequences of behavioural plasticity. Animal Behaviour, 85, 1004–1011. 10.1016/j.anbehav.2012.12.031

[ece36276-bib-0051] Sol, D. , Duncan, R. P. , Blackburn, T. M. , Cassey, P. , & Lefebvre, L. (2005). Big brains, enhanced cognition, and response of birds to novel environments. PNAS, 102, 5460–5465. 10.1073/pnas.0408145102 15784743 PMC556234

[ece36276-bib-0052] Sol, D. , & Lefebvre, L. (2000). Behavioural flexibility predicts invasion success in birds introduced to New Zeland. Oikos, 90, 599–605.

[ece36276-bib-0053] Sol, D. , Lefebvre, L. , & Rodríguez‐Teijeiro, J. D. (2005). Brain size, innovative propensity and migratory behaviour in temperate Palaearctic birds. Proceedings of the Royal Society B, 272, 1433–1441. 10.1098/rspb.2005.3099 16011917 PMC1559823

[ece36276-bib-0054] Sol, D. , Timmermans, S. , & Lefebvre, L. (2002). Behavioural flexibility and invasion success in birds. Animal Behaviour, 63, 495–502. 10.1006/anbe.2001.1953

[ece36276-bib-0056] Taylor, A. H. , Elliffe, D. , Hunt, G. R. , & Gray, R. D. (2010). Complex cognition and behavioural innovation in New Caledonian crows. Proceedings of the Royal Society B, 277, 2637–2643. 10.1098/rspb.2010.0285 20410040 PMC2982037

[ece36276-bib-0057] Vanbereven, S. P. P. , Gebauer, R. , Plichta, R. , Volařík, D. , & Ceulemans, R. (2016). Nutrients and energy in proleptic branches and leaves of poplar under a short‐rotation coppice. Biomass and Bioenergy, 85, 271–277. 10.1016/j.biombioe.2015.12.016

[ece36276-bib-0058] Verovnik, R. , & Konec, M. (2019).*Asellus aquaticus*: A model system for historical biogeography In: White W. B., Culver D. C., & Pipan T. (Eds.), Encyclopedia of caves, Chapter 11 (3rd ed, pp. 76–84). London, UK: Elsevier Academic Press.

[ece36276-bib-0059] Verovnik, R. , Prevorčnik, S. , & Jugovic, J. (2009). Description of a neotype for *Asellus aquaticus* Linné, 1758 (Crustacea: Isopoda: Asellidae), with description of a new subterranean Asellus species from Europe. Zoologischer Anzeiger, 248, 101–111. 10.1016/j.jcz.2009.03.001

[ece36276-bib-0060] Verovnik, R. , Sket, B. , & Trontelj, P. (2004). Phylogeography of subterranean and surface populations of water lice *Asellus aquaticus* (Crustacea: Isopoda). Molecular Ecology, 13, 1519–1532. 10.1111/j.1365-294X.2004.02171.x 15140095

[ece36276-bib-0061] Verovnik, R. , Sket, B. , & Trontelj, P. (2005). The colonization of Europe by the freshwater crustacean *Asellus aquaticus* (Crustacea: Isopoda) proceeded from ancient refugia and was directed by habitat connectivity. Molecular Ecology, 14, 4355–4369. 10.1111/j.1365-294X.2005.02745.x 16313598

[ece36276-bib-0062] West‐Eberhard, M. J. (2003). Developmental plasticity and evolution. New York: Oxford University Press.

[ece36276-bib-0063] Yoshizawa, M. , Robinson, B. G. , Duboué, E. R. , Masek, P. , Jaggard, J. B. , O’Quin, K. E. , … Keene, A. C. (2015). Distinct genetic architecture underlies the emergence of sleep loss and prey‐seeking behavior in the Mexican cavefish. BMC Biology, 13, 15 10.1186/s12915-015-0119-3 25761998 PMC4364459

[ece36276-bib-0064] Zuur, A. , Ieno, E. N. , Walker, N. , Saveliev, A. A. , & Smith, G. M. (2009). Mixed effects models and extensions in ecology with R. New York: Springer‐Verlag.

